# Abdominal Migraine in Adults: A Narrative Review

**DOI:** 10.7759/cureus.85958

**Published:** 2025-06-13

**Authors:** Madunil A Niriella, Hiruni Jayasena, Nilanga Nishad, Indeewari P Wijesingha, Krishanni Prabagar

**Affiliations:** 1 Department of Medicine, Faculty of Medicine, University of Kelaniya, Ragama, LKA; 2 Department of Medicine, General Sir John Kotelawala Defence University, Rathmalana, LKA; 3 Department of Gastroenterology and Hepatology, Addenbrooke's Hospital, Cambridge University Hospitals NHS Foundation Trust, Cambridge, GBR

**Keywords:** abdominal migraine, adults, chronic pain, diagnosis, functional disorders, management

## Abstract

Abdominal migraine (AM) is a debilitating condition characterized by paroxysmal episodes of abdominal pain accompanied by vasomotor symptoms, leading to a significant reduction in quality of life. While more commonly observed in children, AM is rare and less well understood in adults. Diagnostic criteria for adult AM remain poorly defined, and treatment options, both pharmacological and non-pharmacological, have not been extensively evaluated in this population. Pharmacological management may be either prophylactic, aimed at preventing episodes, or abortive, used during acute attacks. Treatment is typically individualized, with a focus on symptom relief and reducing the frequency of attacks. This review summarizes available literature published between 1995 and 2024, with an emphasis on the clinical presentation, diagnosis, and therapeutic strategies for AM in adult patients.

## Introduction and background

Abdominal migraine (AM) is an often overlooked but debilitating condition characterized by recurrent episodes of severe abdominal pain, primarily affecting children yet increasingly recognized in adults.

Abdominal pain is a common yet diagnostically challenging symptom that significantly affects quality of life across all age groups. Population-based and clinical studies from the United States and Europe conducted between 2012 and 2018 estimate that chronic abdominal pain affects approximately 9-15% of children and adolescents [1-3]. Despite extensive investigations, a substantial proportion of these patients have no identifiable organic cause, leading to a diagnosis of functional abdominal pain disorders. These are a group of conditions characterized by chronic abdominal pain without detectable structural or biochemical abnormalities and are diagnosed using established clinical criteria [4]. They include pain-predominant disorders of gut-brain interaction, such as irritable bowel syndrome and functional dyspepsia. Among these, AM is recognized as a distinct subtype, primarily described in pediatric populations, with a reported prevalence ranging from 0.2% to 4.1% [5].

Although historically AM was considered a pediatric condition, recent literature highlights its occurrence in adults as well. A 2023 study emphasizes the emerging trend of AM as a cause of adult abdominal pain and advocates for greater clinical awareness of the condition [6]. Although many children experience a benign course into adulthood without neurological or developmental complications, some continue to suffer persistent symptoms that markedly impair quality of life [7]. Adult patients often undergo numerous investigations and treatments due to the nonspecific nature of symptoms, frequently resulting in frustration and dissatisfaction with healthcare. This highlights the urgent need for increased awareness and understanding of AM in adults to facilitate timely diagnosis and optimize management, potentially preventing poor long-term outcomes.

This narrative review aimed to synthesize current knowledge on AM in adults through a structured examination of the published literature. Our methodology involved an initial screening of article titles and abstracts to identify studies relevant to our research focus, excluding those outside the scope of adult AM. We prioritized peer-reviewed publications that addressed key aspects such as epidemiology, diagnostic criteria, pathophysiology, therapeutic approaches, and clinical management of AM in adults. Rather than cataloging every available study, we focused on distilling key findings from high-quality, representative sources that contribute meaningfully to the understanding of this condition. In cases of discrepancy during article selection, inclusion decisions were made through collaborative discussion and consensus.

A comprehensive literature search was conducted across multiple electronic databases, including PubMed, MEDLINE, Embase, and the Cochrane Library, covering the period from January 1995 to December 2024. The search strategy employed a combination of MeSH terms and relevant keywords, such as “chronic abdominal pain,” “recurrent abdominal pain,” “abdominal migraine,” “adults,” “evaluation,” “diagnosis,” “management,” and “treatment.” Additional relevant studies were identified through manual searches of the reference lists from retrieved articles. Studies were then selected based on their clinical relevance, methodological rigor, and contribution to the thematic focus of the review. Priority was given to systematic reviews, meta-analyses, evidence-based clinical practice guidelines, case series, and case reports. In areas where higher-level evidence was lacking, we also included expert consensus statements and narrative reviews to ensure a comprehensive overview of current perspectives on adult AM.

Despite the comprehensive search extending to late 2024, relatively few recent high-quality studies focusing specifically on adult abdominal migraine were identified. This reflects the emerging nature of research in this field and highlights gaps in current evidence. Consequently, much of the available literature consists of older studies, smaller observational series, and case reports. This limitation highlights the importance of interpreting findings with caution and emphasizes the need for further well-designed, large-scale research to advance understanding of the epidemiology, diagnosis, and management of AM in adults.

## Review

Definitions of abdominal migraine (AM)

AM in children is comprehensively defined under the Rome IV criteria as paroxysmal episodes of intense, acute, periumbilical, midline, or diffuse abdominal pain lasting one hour or more, with episodes occurring weeks to months apart. Each patient may present with a stereotypical pattern of symptoms [8]. The pain is severe enough to interfere with normal daily activities and is associated with two or more of the following: anorexia, nausea, vomiting, headache, photophobia, and pallor. Also, following appropriate medical evaluation, these symptoms should not be attributed to any other underlying medical condition [9].

The International Headache Society (IHS), in its International Classification of Headache Disorders, 3rd edition (ICHD-3), defines AM as an idiopathic disorder predominantly seen in children, characterized by recurrent episodes of moderate to severe midline abdominal pain accompanied by vasomotor symptoms, nausea, and vomiting. These episodes last between 2 to 72 hours and are interspersed with symptom-free periods. Notably, headache is absent during these abdominal episodes [10]. A thorough history and physical examination should reveal no signs of gastrointestinal or renal disease, or such conditions should be excluded through appropriate investigations. A major limitation of both the Rome IV and ICHD-3 classifications is that they are restricted to the pediatric population.

Table 1 summarizes the diagnostic criteria proposed by the Rome IV and ICHD-3 classifications for AM. It is important to note that AM is included only in the pediatric Rome IV criteria for functional gastrointestinal disorders and is not recognized in the adult Rome IV criteria.

**Table 1:** Comparison of Rome IV vs ICHD III Diagnostic Criteria This table is an original work based on information derived from references [8, 10].

Feature	ROME IV [8]	IHS / ICHD-3 [10]
Source	Rome Foundation's Diagnostic Criteria for Functional Gastrointestinal Disorders	International Classification of Headache Disorders, 3rd edition (ICHD-3)
Primary Focus	Functional gastrointestinal disorders	Headache disorders
Definition	A functional gastrointestinal disorder characterized by recurrent episodes of intense, acute periumbilical pain	Recurrent attacks of moderate to severe midline abdominal pain, associated with vasomotor symptoms, nausea, and vomiting
Pain Location	Periumbilical	Midline abdominal
Pain Intensity	Intense, acute	Moderate to severe
Duration	≥1 hour	2-72 hours (untreated or unsuccessfully treated)
Associated Symptoms	At least 2 of: anorexia, nausea, vomiting, headache, photophobia, pallor	At least 2 of: anorexia, nausea, vomiting, pallor, photophobia
Vasomotor Symptoms	Not specifically mentioned	Explicitly mentioned
Headache	Listed as a possible associated symptom	Not specifically listed
Return to Baseline Health	Between episodes	Between attacks
Exclusion of Other Causes	Yes, after appropriate evaluation	Yes, not better accounted for by another disorder
Chronicity Requirement	Must fulfill criteria for at least 6 months prior to diagnosis	No specific chronicity requirement
Frequency Requirement	≥2 times in the preceding 6 months	At least 5 attacks
Age of Onset	Not specified	Usually begins in childhood (before age 12)
Family History	Not part of diagnostic criteria	Often a positive family history of migraine
Relation to Migraine	Not part of diagnostic criteria	Considered a childhood periodic syndrome that is commonly a precursor of migraine

Pathophysiology

Although the exact pathophysiology of AM remains unclear, several contributing mechanisms have been proposed. These include visceral hypersensitivity, altered gut motility - now referred to as “Disorders of Gut-Brain Interaction (DGBI)” - and psychological factors that influence the hypothalamic-pituitary-adrenal axis [11,12].

Central to these mechanisms is the gut-brain axis, a bidirectional communication system between the gastrointestinal (GI) tract and the central nervous system (CNS), which plays a critical role in maintaining homeostasis and modulating gut function. Serotonin (5-HT), a key neurotransmitter in the regulation of this axis, is believed to play a pivotal role in the pathogenesis of AM. Fluctuations in serotonin levels can influence neuronal excitability and vascular tone, potentially contributing to the abdominal pain and discomfort seen in functional GI disorders [13,14].

Psychological factors may also play a significant role in AM. Adverse life experiences such as abuse and chronic stress are recognized risk factors. Furthermore, children with DGBIs are more likely to exhibit mental health conditions such as anxiety and depression, which may further exacerbate AM symptoms [1-3,15].

Additionally, it is hypothesized that AM shares pathophysiological mechanisms with traditional migraine. Activation of the trigeminovascular system can lead to neurogenic inflammation and the release of proinflammatory peptides such as substance P, vasoactive intestinal peptide (VIP), and calcitonin gene-related peptide (CGRP), all of which are implicated in abdominal pain generation [16,17]. CGRP, in particular, is a potent vasodilator expressed in the enteric nervous system and has been shown to induce severe gastrointestinal symptoms when administered through prolonged infusion [18].

Epidemiology

The true prevalence and clinical outcomes of AM in adults remain unclear due to limited and conflicting literature. However, among children with recurrent abdominal pain, the estimated prevalence of AM ranges from 2.4% to 4.1%, with a mean age of onset around seven years [19].

AM can occur in both sexes and has been most commonly observed during the second and third decades of life. In children, there is a female predominance, with a reported female-to-male ratio of 1.6:1 [20]. A strong family history of migraine is also commonly noted in affected individuals [21].

There is a clear need for larger, well-designed studies to accurately determine the prevalence, clinical features, natural history, and long-term prognosis of AM in adults due to conflicting evidence. A longitudinal study from the UK that followed 54 children diagnosed with AM found that the condition resolved in approximately 61% of participants over an 8-10-year period. However, a significant proportion continued to experience recurrent abdominal pain during migraine attacks into adulthood [22]. In contrast, another study reported that only 1.5% of adults experienced recurrent abdominal pain associated with migraine attacks [23].

Clinical presentation

Adult patients with AM often experience symptoms similar to those observed in children, most notably episodes of chronic abdominal pain. A distinguishing characteristic of AM is its paroxysmal and episodic nature, which sets it apart from many other gastrointestinal disorders that typically present with chronic, more frequent symptoms, often occurring 2 to 5 times per week [3].

AM also shares clinical features with several adult-onset conditions, including cyclic vomiting syndrome (CVS), irritable bowel syndrome (IBS), cannabinoid hyperemesis syndrome, narcotic bowel syndrome, functional dyspepsia (FD), and centrally mediated abdominal pain syndrome (CAPS). CVS, like AM, presents with recurrent episodes separated by symptom-free intervals; however, CVS is often marked by more severe vomiting and dehydration. IBS is characterized by abdominal pain, bloating, and altered bowel habits, but the pain is generally less intense and more continuous compared to AM’s episodic attacks. Cannabinoid hyperemesis syndrome can mimic AM but is typically linked to chronic cannabis use, which should be carefully assessed during history taking. Narcotic bowel syndrome is driven by opioid use, with symptoms worsening with continued consumption, unlike AM, which occurs independently of opioid use and manifests as discrete episodes. FD is associated with persistent epigastric discomfort, early satiety, and postprandial bloating; however, its symptoms are usually milder and less episodic than those of AM. CAPS is characterized by continuous and widespread abdominal pain without symptom-free intervals, in contrast to the well-demarcated episodic pain seen in AM. Therefore, while AM is less common than these conditions in adults, it should remain a differential diagnosis in patients presenting with episodic abdominal pain, especially after excluding other potential causes [24,25].

AM, CVS, and migraine headaches often overlap and may evolve throughout a patient’s life. Children with AM may later develop migraines or CVS, and vice versa. For example, patients with CVS may experience abdominal pain, while those with AM may present with vomiting and migraine headaches [25]. This overlap complicates classification and contributes to inconsistencies, thereby limiting a comprehensive understanding of these disorders.

Table 2 summarizes the clinical characteristics of AM reported in adult cases.

**Table 2:** Clinical Characteristics of Abdominal Migraine (AM) of the Reported Cases in the Literature This table is an original work based on information in references [6, 9, 21, 25-33]. GERD: Gastroesophageal reflux disease; NSAID: Nonsteroidal anti-inflammatory drugs

Author	Sex	Age	Symptoms	Duration	Co-morbidities	Time to diagnosis from onset	History of migraine	Family history	Non-responding Rx	Treatment (abortive)	Treatment (prophylaxis)
Woodruff et al. [6]	F	32	Abdominal pain, nausea, vomiting	3-4 days	Type 1 DM, GERD	5-6 years	None noted	Yes	Sumatriptan (oral)	-	Topiramate
Evans and Whyte [9]	M	52	Shoulder & abdominal pain, autonomic symptoms	12-13 hrs	Multiple	3 years	Yes	None noted	Multiple drugs	Eletriptan, Prochlorperazine	-
Evans and Whyte [9]	M	53	Shoulder & abdominal pain, autonomic symptoms	12-13 hrs	Multiple	4 years	Yes	None noted	Multiple drugs	Eletriptan, Prochlorperazine	-
Evans and Whyte [9]	M	54	Shoulder & abdominal pain, autonomic symptoms	12-13 hrs	Multiple	5 years	Yes	None noted	Multiple drugs	Eletriptan, Prochlorperazine	-
Kunishi et al. [21]	F	52	Abdominal pain, nausea, vomiting	Half a day	Infectious enteritis	1 month	Yes	Yes	Multiple drugs	Loxoprofen	Lomerizine
d'Onofrio et al. [25]	F	23	Abdominal pain, autonomic symptoms	6 hrs, 4-5/month	Ovarian cysts	7 years	No	Yes (mother, sister)	NSAIDs, Sumatriptan	-	Pizotifen
Hamed [26]	M	20	Abdominal colic, autonomic symptoms	Weekly, up to 72 hrs	None	10 years	No	Yes	Colchicine	Eletriptan	Valproate
Roberts and deShazo [27]	F	48	Abdominal pain, nausea, vomiting	Hours, >1/month	Classic migraine	2 years	Yes	Yes	Not mentioned	Rizatriptan	Topiramate
Roberts and deShazo [27]	F	24	Severe abdominal pain, autonomic symptoms	Hours to 2 days	Surgery for intussusception	5 years	None noted	None noted	Not mentioned	-	Topiramate
Dees et al. [28]	M	59	Left lower quadrant pain	Hours to months	Multiple	-	Yes	Yes	-	Divalproex	-
Rasmussen and Nøjgaard [29]	F	25	Nausea, vomiting, abdominal pain	2 Years	None	2 years	Yes	Yes	Multiple drugs	Propranolol	-
Newman and Newman [30]	M	26	Abdominal pain, nausea, vomiting, pallor	Initially weekly, later daily	None	4 years	No	Yes	Multiple drugs	Sumatriptan	Topiramate, Verapamil
Pryszmont [31]	F	35	Nocturnal abdominal pain, nausea	4 years	None	4 years	Yes	Yes	-	Pizotifen	Multiple drugs
Karmali and Hall-Wurst [32]	F	58	Abdominal pain, coffee ground vomitus	7 months	Ischemic heart disease	1 year	No	None noted	Multiple drugs	Prochlorperazine	-
Cervellin and Lippi [33]	F	30	Periumbilical pain, vomiting	12 hrs, 4-5/month	None	15 years	None noted	None noted	-	Multiple drugs	-

As seen above, pain is the hallmark symptom of AM and can occur at any site within the abdomen [21]. It is most commonly experienced in the periumbilical, epigastric, or midline regions, though some patients report diffuse abdominal discomfort. The severity of the pain varies widely, from dull, crampy sensations to moderate or severe, intense episodes. In rare cases, the pain can be so severe that it leads to transient loss of consciousness. These episodes can significantly impair daily functioning and reduce overall quality of life. The pain is typically not related to meals or a specific time of day, and its duration can range from 2 to 72 hours. Patients are usually asymptomatic between attacks, with no residual symptoms [26,27].

In addition to abdominal pain, patients may also experience nonspecific autonomic and vasomotor symptoms such as pallor, nausea, vomiting, and anorexia. Some individuals report sensitivity to light and noise, similar to symptoms observed in migraine sufferers [27,28]. Due to the nonspecific, relapsing-remitting nature of these symptoms, diagnosis is often significantly delayed, ranging from several months to 10-15 years in many cases.

Diagnostic evaluation

Due to its unique and variable presentation, AM is often misdiagnosed or overlooked. Currently, there are no established guidelines for diagnosing or managing AM in adults. Therefore, diagnosis should be based on a thorough medical history, physical examination, and appropriate investigations.

When taking the patient’s history, it is essential to assess for the diagnostic criteria outlined by the International Headache Society (IHS) or ROME IV criteria (Table 1) for children. While the criteria in Table 1 may assist clinicians in recognizing AM and support research efforts, they have not been validated in adults. Thus, they should not be solely relied upon for a positive diagnosis without first ruling out organic causes. Abdominal migraine remains a diagnosis of exclusion. Given the overlap of symptoms with other conditions, excluding alternative diagnoses is also crucial.

Additionally, patient history should exclude “alarm symptoms” such as unexplained weight loss, melena, rectal bleeding, or altered bowel habits, which may indicate more serious underlying pathology requiring further invasive investigations [29]. Other medical conditions, including renal or vascular diseases that could explain the symptoms, must also be ruled out before confirming the diagnosis of AM, as emphasized in the ROME IV and IHS guidelines for children [8,10]. A positive family history of migraine is also common, especially among female first-degree relatives, which should be evaluated in the history. Some of these patients also report that typical migraine triggers, such as alcohol and stress, precipitate their abdominal migraine episodes hence they should be inquired [20]. AM should be considered in the differential diagnosis of chronic, recurrent abdominal pain, especially in patients with a personal or family history of migraine. Importantly, in adults, AM may present in the absence of an accompanying headache.

If necessary, investigations can be done. Non-invasive tests precede serological tests such as full blood count (FBC), C-reactive protein (CRP), to rule out infective causes. Moreover, stool for calprotectin and/or occult blood can also be conducted to rule out inflammatory and neoplastic causes. Imaging of the abdomen with ultrasound (US) or computer tomography (CT) would aid in ruling out common abdominal pathologies such as gallstones, appendicitis, pancreatitis or intraabdominal malignancies. Finally, invasive testing with endoscopy could be done to rule out the presence of common gastrointestinal conditions such as peptic ulcer disease and gastritis. The presence of other functional abdominal pain disorders (IBS and FD) must also be considered after assessment of diet and psychological status.

Management

The approach to treatment depends on the patient’s age, symptom severity, and individual response to therapy. Patient education and reassurance are central to management and represent the first-line treatment. The main goals are to reduce the frequency of attacks and prevent debilitating episodes. Educating patients about AM, reassuring them that serious pathology is absent despite their symptoms, and emphasizing the likelihood of symptom improvement with age are essential. An observational study found that up to 60% of patients with parents affected by AM felt relieved after gaining an understanding of the condition and its symptoms [20,34].

Diet modification is also used as a treatment and appears to play a significant role in managing AM in children, up to 93% report at least one food type that worsens their gastrointestinal symptoms [35,36]. A randomized controlled trial involving 52 children showed that 50% of those in the fiber-supplemented group experienced a 50% reduction in abdominal pain episodes, compared to 27% in controls [37]. Similarly, reducing lactose intake has been associated with fewer AM episodes in children [38]. Some studies also suggest that insufficient fiber intake during childhood may contribute to abdominal pain [37,39]. However, the effectiveness of these dietary interventions in adults with AM remains unclear. Most adult studies focus on IBS, with very few including patients diagnosed with AM. The efficacy of low FODMAP or gluten-free diets, which are primarily used in IBS, has not been adequately studied in adults with AM. Although AM shares pathophysiological similarities with migraine, and dietary triggers are recognized in migraine, there is no clear evidence supporting dietary modification as a treatment in adults with AM. While pediatricians often attribute migraine symptoms to diet, definitive evidence of benefit from dietary changes in adults with AM is lacking.

Cognitive behavioral therapy (CBT) is another treatment modality used in treatment. It has been extensively shown to be effective in functional abdominal pain disorders such as IBS and functional dyspepsia, and has also been used successfully in migraine [40]. However, its role in adult AM has yet to be established. Other therapies, including yoga and hypnotherapy, which have shown benefit in some functional abdominal pain syndromes, may also be helpful, although further research is needed to confirm their efficacy in adult AM. However, simple measures used in acute migraine, such as resting in a dark, quiet room with analgesics, have been reported to relieve acute AM symptoms in over 80% of patients during attacks [28].

While non-pharmacological approaches are typically first-line, many patients seek pharmacological treatment to reduce attack frequency and achieve remission. However, evidence supporting pharmacological treatments in adult AM is limited. Drug therapy is usually reserved for those with severe, recurrent symptoms that significantly impair quality of life. Options include medications for acute symptom relief as well as prophylactic agents. Various drug classes have been used prophylactically in AM, often alongside conventional migraine therapies. These include antiepileptic drugs such as valproate and topiramate; antidepressants like amitriptyline; serotonin antagonists such as cyproheptadine; beta-blockers including nadolol and propranolol; and calcium channel blockers such as verapamil and lomerizine. Pizotifen also shows promising evidence as a prophylactic agent, but mostly in children with AM [41], but larger randomized controlled trials are needed to confirm its benefit in adults. Similarly, a pediatric case series reported the effectiveness of intranasal sumatriptan in patients with migraine and abdominal pain [42], but no studies have been done in adults. However, triptan has been studied in adults with AM. A small 2012 study suggested that triptans could serve diagnostically in adults to distinguish AM from other chronic abdominal pain syndromes, as symptoms resolved in all but one patient following triptan administration. Triptans are generally effective during acute attacks, especially when taken early and often combined with antiemetics [28]. Although most medications are administered orally, subcutaneous delivery is preferred in severe cases complicated by nausea and vomiting that limit oral intake. The role of simple analgesics such as NSAIDs and paracetamol requires further investigation; most reported responders have been children with AM [43,44].

Table 3 summarizes the characteristic features of AM discussed in this review.

**Table 3:** Key Features of Adult Abdominal Migraine: Overview of Multiple Case Reports This table is an original work summarising multiple case reports based on references [6, 9, 20, 21-25, 27-29, 32]

Patient Characteristic	Description
Demographics	18 cases (11 females, 7 males); Age range: 20-59 years
Symptoms	Abdominal pain (all cases) Locations: periumbilical, left lower quadrant, epigastric Autonomic symptoms: nausea, vomiting, pallor, photophobia
Pain Characteristics	Duration: 30 mins to several days Frequency: daily to monthly
Co-morbidities	Various, including: migraines, gastrointestinal disorder, cardiovascular diseases
Migraine History	Personal history: 12/18 (67%) Family history: 10/18 (56%)
Treatment: Abortive	NSAIDs, Triptans (Sumatriptan, Eletriptan, Rizatriptan), Antiemetics
Treatment: Prophylaxis	Topiramate (5 cases), Others: Pizotifen, anticonvulsants, beta-blockers, calcium channel blockers
Treatment Response	Many didn't respond to initial treatments, requiring multiple drug trials
Time to Diagnosis	1-16 years from symptom onset
Key Insights	Variable presentation, Strong association with classical migraine, Management challenges, Underrecognized in adults

## Conclusions

The true prevalence of AM in adults remains uncertain due to frequent misdiagnosis and underdiagnosis. Many adult patients undergo extensive investigations and multiple consultations before receiving an accurate diagnosis. There is a clear need for further research to better characterize the clinical presentation, refine diagnostic criteria, and evaluate treatment options specifically for adults, as most current evidence is extrapolated from pediatric studies.

Based on this review, clinicians should consider AM in adults presenting with recurrent, paroxysmal abdominal pain accompanied by two or more associated symptoms such as anorexia, headache, vomiting, photophobia, or pallor, especially when symptoms persist for years. A positive family history and the use of Rome IV and ICHD-3 diagnostic criteria may aid in diagnosis. Although CBT and dietary interventions show promise, their effectiveness in adults requires further study. Additionally, case reports suggest a poor response to simple analgesics and other medications, highlighting the urgent need for well-designed clinical trials to re-evaluate pharmacological treatments for adult AM.
